# Origin of Intra-annual Density Fluctuations in a Semi-arid Area of Northwestern China

**DOI:** 10.3389/fpls.2021.777753

**Published:** 2021-11-22

**Authors:** Jiani Gao, Sergio Rossi, Bao Yang

**Affiliations:** ^1^Key Laboratory of Desert and Desertification, Northwest Institute of Eco-Environment and Resources, Chinese Academy of Sciences, Lanzhou, China; ^2^College of Resources and Environment, University of Chinese Academy of Sciences, Beijing, China; ^3^Département des Sciences Fondamentales, Université du Quebec à Chicoutimi, Chicoutimi, QC, Canada; ^4^Key Laboratory of Vegetation Restoration and Management of Degraded Ecosystems, Guangdong Provincial Key Laboratory of Applied Botany, South China Botanical Garden, Chinese Academy of Sciences, Guangzhou, China; ^5^Center for Excellence in Tibetan Plateau Earth Sciences, Chinese Academy of Sciences, Beijing, China; ^6^Qinghai Research Center of Qilian Mountain National Park, Academy of Plateau Science and Sustainability and Qinghai Normal University, Xining, China

**Keywords:** water availability, IADF, xylogenesis, cambial activity, Chinese pine, cell enlargement

## Abstract

Intra-annual density fluctuation (IADF) is a structural modification of the tree ring in response to fluctuations in the weather. The expected changes in monsoon flow would lead to heterogeneous moisture conditions during the growing season and increase the occurrence of IADF in trees of the arid ecosystems of continental Asia. To reveal the timings and physiological mechanisms behind IADF formation, we monitored cambial activity and wood formation in Chinese pine (*Pinus tabuliformis*) during 2017–2019 at three sites in semi-arid China. We compared the dynamics of xylem formation under a drought event, testing the hypothesis that drought affects the process of cell enlargement and thus induces the production of IADF. Wood microcores collected weekly from April to October were used for anatomical analyses to estimate the timings of cambial activity, and the phases of enlargement, wall thickening, and lignification of the xylem. The first cells started enlargement from late April to early May. The last latewood cells completed differentiation in mid-September. Trees produced IADF in 2018. During that year, a drought in June limited cell production in the cambium, only 36% of the xylem cells being formed in IADF trees, compared to 68% in normal tree rings. IADF cells enlarged under drought in early July and started wall thickening during the rainfall events of late July. The drought restricted cell enlargement and affected wall thickening, resulting in narrow cells with wide walls. Cambium and cell enlargement recovered from the abundant rainfall, producing a new layer with large earlywood tracheids. IADF is a specific adaptation of trees to cope with water deficit events occurring during xylem formation. Our findings confirmed the hypothesis that the June-July drought induces latewood-like IADFs by limiting the process of cell enlargement in the xylem. Our finding suggests a higher occurrence of IADF in trees of arid and semi-arid climates of continental Asia if the changes to monsoon flows result in more frequent drought events during the earlywood formation in June.

## Introduction

Intra-annual density fluctuation (IADF) is a structural modification of the tree ring involving an abrupt change in wood density (Battipaglia et al., 2016), producing a cell layer similar to the boundary of a true tree ring. IADF exhibit either latewood-like cells with thicker cell wall and narrower lumen area in earlywood intra-annual density fluctuations (*E*-IADFs), or earlywood-like cells with larger lumina and thinner walls in latewood intra-annual density fluctuation (*L*-IADFs; Campelo et al., 2007). Large tracheids are the key for efficient water conduction but are more vulnerable to cavitation and embolism. Thick cell walls contribute to the increase of wood density (Beeckman, 2016). IADF represents the ability of a species to adapt to the substantial changes in the growing conditions according to changes in xylogenesis, i.e., cambial activity or cell differentiation or both (Eilmann et al., 2011; Deslauriers et al., 2016; De Micco et al., 2016a). The morphology of IADF cells affects the hydraulic properties of wood and tree survival. In addition, an abrupt modification of the tree-ring structure due to IADF affects the physical or mechanical properties of the xylem and reduces wood quality and its potential use (Olivar et al., 2015).

Intra-annual density fluctuations have been studied mainly in Mediterranean ecosystems, characterized by a long growing season with a hot and dry summer, inducing bimodal growth patterns in most conifers. Trees in Mediterranean ecosystems generally exhibit *L*-IADFs occurring after the summer drought, at the beginning of the autumnal rainfall (Battipaglia et al., 2010; De Micco et al., 2016b; Balzano et al., 2018). *E*-IADFs are observed in temperate forests, as a result of latewood cells developed within earlywood or at the transition between earlywood and latewood (Edmondson, 2010; Rozas et al., 2011; Gao et al., 2021). *E*-IADF is linked to drought conditions during the summer. IADFs occur in other environments such as tropical and boreal forests (Marchand and Filion, 2012; Palakit et al., 2015; Venegas-González et al., 2015). Despite the wide literature on *L*-IADF, knowledge on the mechanisms of the formation on *E*-IADF remains unclear in ecosystems outside the Mediterranean area, especially in arid inland areas. Based on the available literature, IADF is less investigated in China (Zhang et al., 2020; Gao et al., 2021).

Precipitation and temperature are the climatic factors most frequently considered in relation to IADF, although late frosts and defoliation may be involved in its formation (De Micco et al., 2016b). The study indicated that 72% of the years showing IADF in the tree ring of black pine had low precipitations in May (Wimmer et al., 2000). The frequency of IADFs varies greatly among trees with different species, ages, and tree ring widths. Different species show a different aptitude to form IADFs (Balzano et al., 2019). It is also suggested that younger trees with wider tree rings are more prone to form IADF than older trees with narrower rings (Schweingruber, 1996). Knowledge on the occurrence, frequency, and mechanisms of the production on IADF remains scarce and deserve more attention. Detailed analyses of xylem phenology at high time resolution can be a tool to characterize IADF phenology and its environment drivers.

The rise in temperature worldwide, associated with local reduction in precipitation, is expected to increase frequency and severity of warming-induced drought (IPCC. Climate Change, 2014; Wu et al., 2018). Vegetation and fertility of Eastern Asia benefits from the wet spring and summer resulting from precipitation due to the periodic monsoons (Ding et al., 2018). The asymmetric changes in land and ocean temperatures are expected to weaken the monsoon flows, resulting in a drier climate, with serious consequences for the arid Asian regions (Huang et al., 2017). The fluctuations of monsoon intensity would lead to diversified water vapor patterns and frequent extreme climate events, making trees growing in this area experience a more complicated hydrothermal environment. Severe drought events and fluctuations in moisture conditions affect stem growth and increase the production of abnormal tree ring structures and IADFs (Cuny and Rathgeber, 2016). This region is therefore an ideal location to investigate the responses of xylogenesis to drought.

In this study, we collected samples in Chinese pine (*Pinus tabuliformis*) in three sites located in the semi-arid region lying between the East Asian monsoon zone and the arid region of Northwestern China and investigated the dynamics of xylogenesis and IADF formation. The objective of this study was to (i) assess the phenology of IADF and (ii) investigate the climatic drivers of IADF production. We tested the hypothesis that summer drought affects the process of cell enlargement and thus induces IADF.

## Materials and Methods

### Study Site and Tree Selection

**Figure 1:**
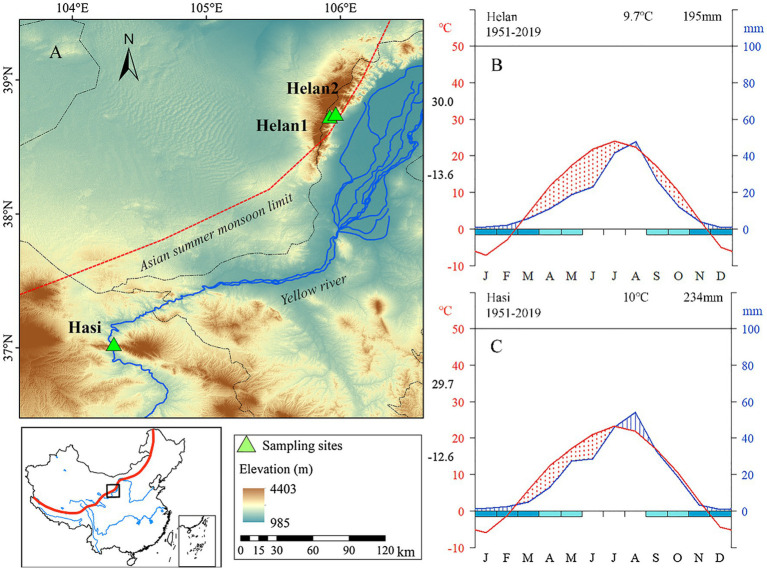
**(A)** Map of sampling sites, and **(B,C)** Walter and Lieth climatic diagram at Helan and Hasi during 1951–2019. Red line and blue line represent the monthly mean temperature and precipitation. Values on the left axis are the average maximum temperature of the warmest month and average minimum temperature of the coldest month. The upper right of the diagram shows an annual mean temperature and annual total precipitation.

We selected three sites in a semi-arid forest in Northwestern China (Figure 1A). Two sites (named as Helan1 and Helan2) were located at two altitudes (2010ma.s.l. and 2,330ma.s.l.) at the Helan Mountain national forest park in Ningxia (38°44′ N, 105°54′ E). The third site (named as Hasi) was located 400km away, on Hasi Mountain (37°03′ N, 104°31′ E, 2410ma.s.l.). This study area forms the boundary between the arid region in the Northwest and the East Asian monsoon zone. Chinese pine (*Pinus tabuliformis*) is one of the dominant tree species growing at the northwestern boundary of its distribution. The long-term climate recorded by the national weather stations during 1951–2019 showed similar mean annual temperature between the two regions, with 9.7 and 10°C in Helan and Hasi, respectively (Figures 1B,C). Total precipitation was 195 and 234mm, with 65 and 55% of the rain falling in June–August, at Helan and Hasi, respectively. The climate is dry in winter and generally wet from spring to autumn. Hasi receives more precipitation in late spring and summer compared with Helan.

Sixteen dominant and healthy Chinese pines were chosen (eight trees per site at Helan1 and Helan2, 10 trees at Hasi). Trees at Hasi were bigger, i.e., larger and taller, and older than those at the other two sites (Table 1). The younger trees (70years old) were located at Helan2.

**Table 1:** Characteristics of the trees sampled in three sites in a semi-arid region of Northwestern China.

	DBH (cm)	Height (m)	Age (years)
Helan1	72±9	7.6±1.8	103±7
Helan2	72±12	10.5±1.5	70±10
Hasi	105±23	12±4	93±25

### Meteorological Data

Three automated weather stations were installed at each site to measure air temperature and precipitation every 30min, and daily means were calculated from the half-an-hour time series. To quantify drought severity, the standardized precipitation evapotranspiration index (SPEI) was calculated according to the Hargreaves equation, as a monthly difference between precipitation and potential evapotranspiration, using “SPEI” package (Beguería et al., 2017) in R environment (R Core Team, 2017).

### Xylem Collection and Observation

Wood samples were collected in 2017–2019 weekly, at breast height (1.3m above ground), from April to September, the main growing season of Chinese pine (Zeng et al., 2018) using a Trephor (Rossi et al., 2006a), and stored in 50% ethanol solution. Microcores were dehydrated through successive immersions in ethanol solutions and dewaxing agent, embedded in paraffin, cut in sections (8–12μm thickness) using a rotary microtome, and then stained with a water solution of safranin and astral blue. Sections were examined under visible and polarized light at 200–400 ×magnifications to discriminate cells in different stages of xylem differentiation. The number of cells in the cambial zone, enlargement, secondary wall thickening, and mature xylem cells were counted along three radial files. Cambial cells were flat with thin primary walls. Enlarging cells were at least twice the radial length of cambial cells, with thin cell walls. In spring, xylogenesis was considered to have started when at least one horizontal row of enlarging cells was observed (Rossi et al., 2006b). Cell wall thickening and mature cells were blue or light red and dark red, respectively, with thick walls and showed birefringence under polarized light. We consider that xylem formation was complete when all cells of the tree ring were mature.

### IADFs Identification and Statistics

We recorded the proportion of trees with IADFs in all sampling trees in each site and year; we compared difference in monthly air temperature, precipitation, and SPEI using mixed models to assess which factor affected IADFs occurrence. Year and site were considered as random effects in the model. To identify the timings of IADFs formation, the number of cells in earlywood, IADFs, and latewood were counted and reported in the form of proportion. Tracheids were classified as earlywood or latewood according to the Mork’s formula, which classifies all cells with lumen areas of less than twice the thickness of a double cell wall as latewood (Denne, 1988). The cumulative proportion of (i) mature cells, (ii) mature cell and wall thickening cells, and (iii) total xylem cells (mature, wall thickening, and enlarging cells) in the tree ring for each day in 2018 were estimated using the Gompertz function. The function is defined as:


$$Y\left(t\right)=Ae^{-e^{\beta -kt}}$$

where *Y*(*t*) is the cumulative proportion of cells at time t; *A* is the upper asymptote parameter; *β* is the x-axis placement parameter; and *k* is the rate of change parameter. The cumulative proportion of mature cells, mature cell and wall thickening cells, and total xylem cells represented the timings when the cells produced by the cambium underwent enlargement and wall thickening (Rossi et al., 2003).

## Results

### IADF Occurrence

**Figure 2:**
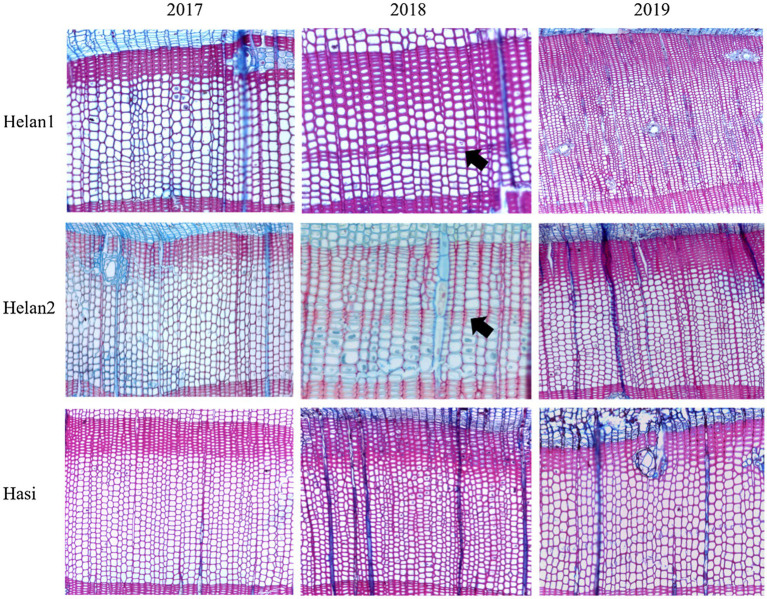
Wood anatomy in tree-rings sampled at three sites during 2017–2019 in a semi-arid region of Northwestern China. Arrows indicate the IADF.

The sections of all sampled trees during 3years were identified to investigate the occurrence of IADFs. No IADFs were observed at three sites in 2017 and 2019 (Table 2). A normal tree ring structure exhibits large, thin-walled earlywood cells, followed by small, heavy-walled latewood cells (Figure 2). E-IADFs were observed in earlywood of all trees from Helan1 and Helan2 sampled in 2018. E-IADF appeared in the form of narrow cells with thick walls within the earlywood zone. No IADF was observed at Hasi.

**Table 2:** Occurrence of Intra-annual density fluctuations (IADFs) of the trees sampled in three sites in a semi-arid region of Northwestern China. Values are reported as trees showing IADFs on total sampled trees.

	2017	2018	2019
Helan1	0/8	8/8	0/8
Helan2	0/8	8/8	0/8
Hasi	0/10	0/10	0/10

**Figure 3:**
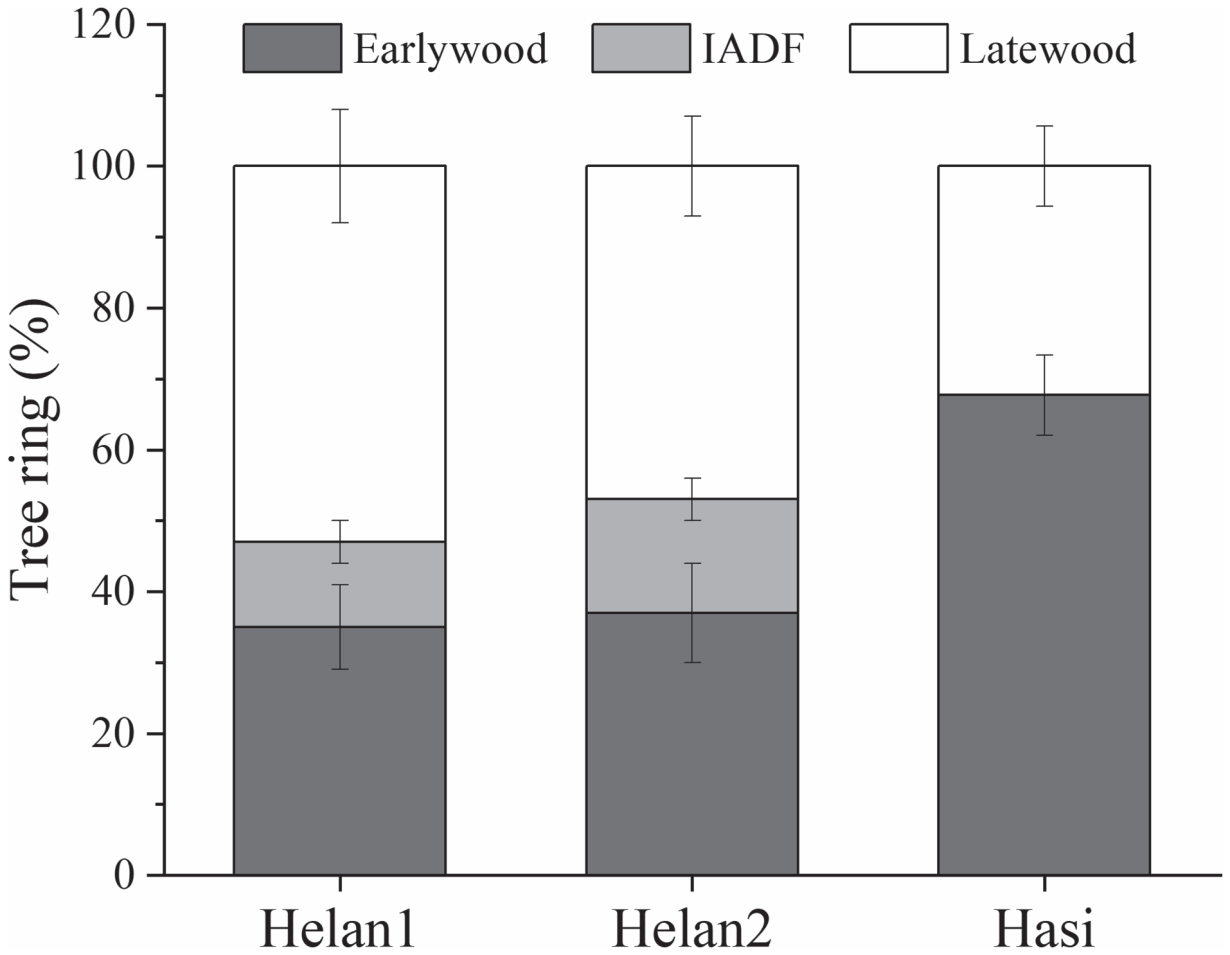
Proportion of earlywood, IADF, and latewood in tree ring in 2018 at three sites in a semi-arid region of Northwestern China.

In 2018, 35% of xylem cells were classified as earlywood at Helan1 and Helan2 (Figure 3). IADFs accounted for about 12 and 16% of tree ring at Helan1 and Helan2, and 50% of the tree ring was latewood at the two sites. At Hasi, 68% of the tree ring was earlywood, and 32% was latewood.

### Comparing Climate Between Years

**Figure 4:**
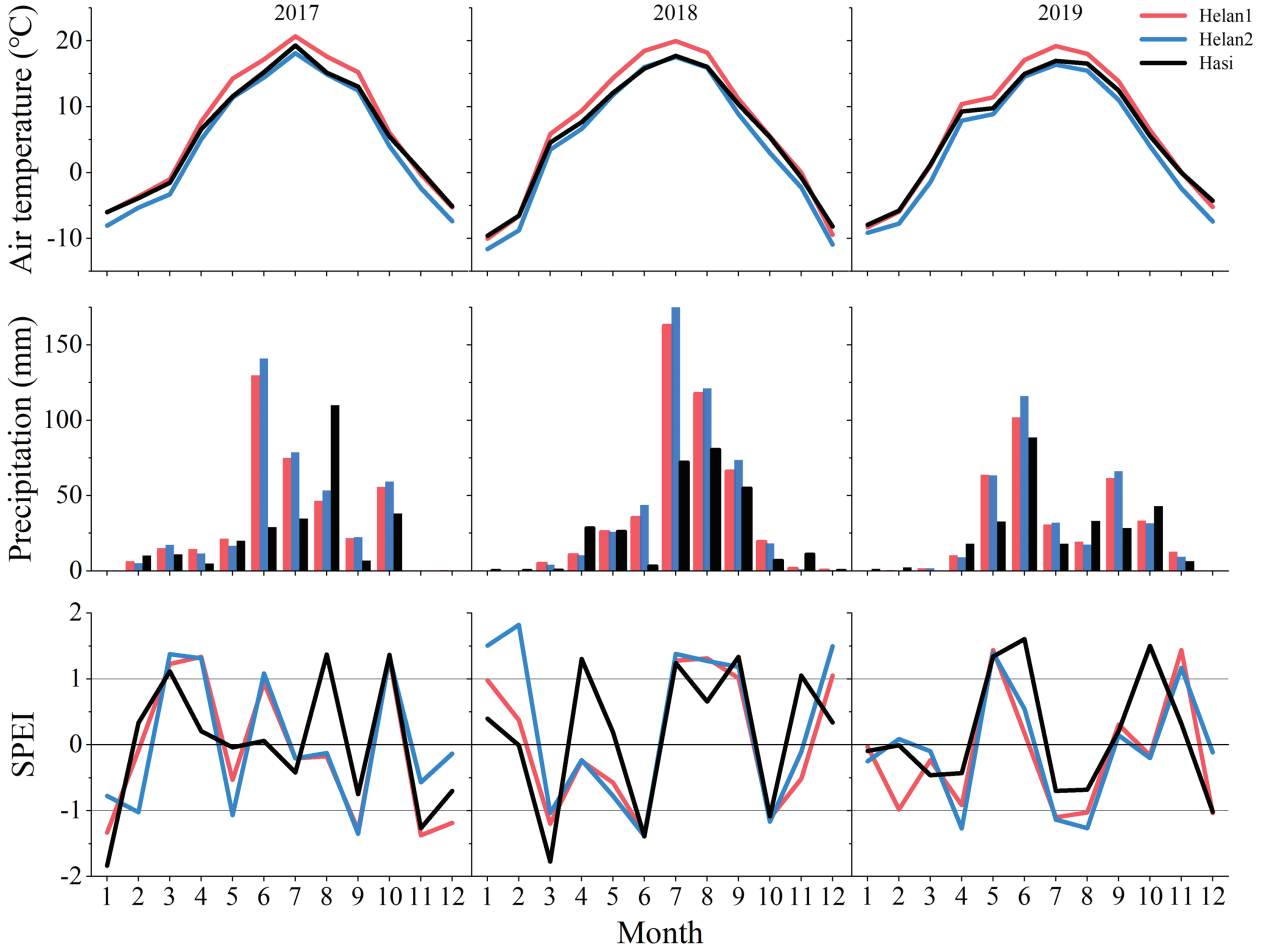
Monthly mean air temperature, precipitation and SPEI at three sites during 2017–2019.

The years 2017 and 2019 were warmer than 2018. A lower precipitation was recorded in 2019 compared to the other 2years (Figure 4). The mean air temperature was 6.3°C in March and April 2018, which was 4°C and 1.6°C higher than that in 2017 and 2019, respectively. Precipitation of June was lower in 2018 (27mm) compared to 2017 (100mm) and 2019 (102mm). However, rain was more abundant in July and August in 2018 compared to the same period in 2017 and 2019. As a consequence, the climate in June was dry in 2018, with SPEI being <−1. In June 2017 and 2019, SPEI indicated wet conditions. July and August were wetter in 2018 compared to 2017 and 2019. Overall, SPEI of April and May 2018 was higher at Hasi (1.3 and 0.19) compared to Helan1 (−0.25 and−0.58) and Helan2 (−0.23 and−0.78).

**Figure 5:**
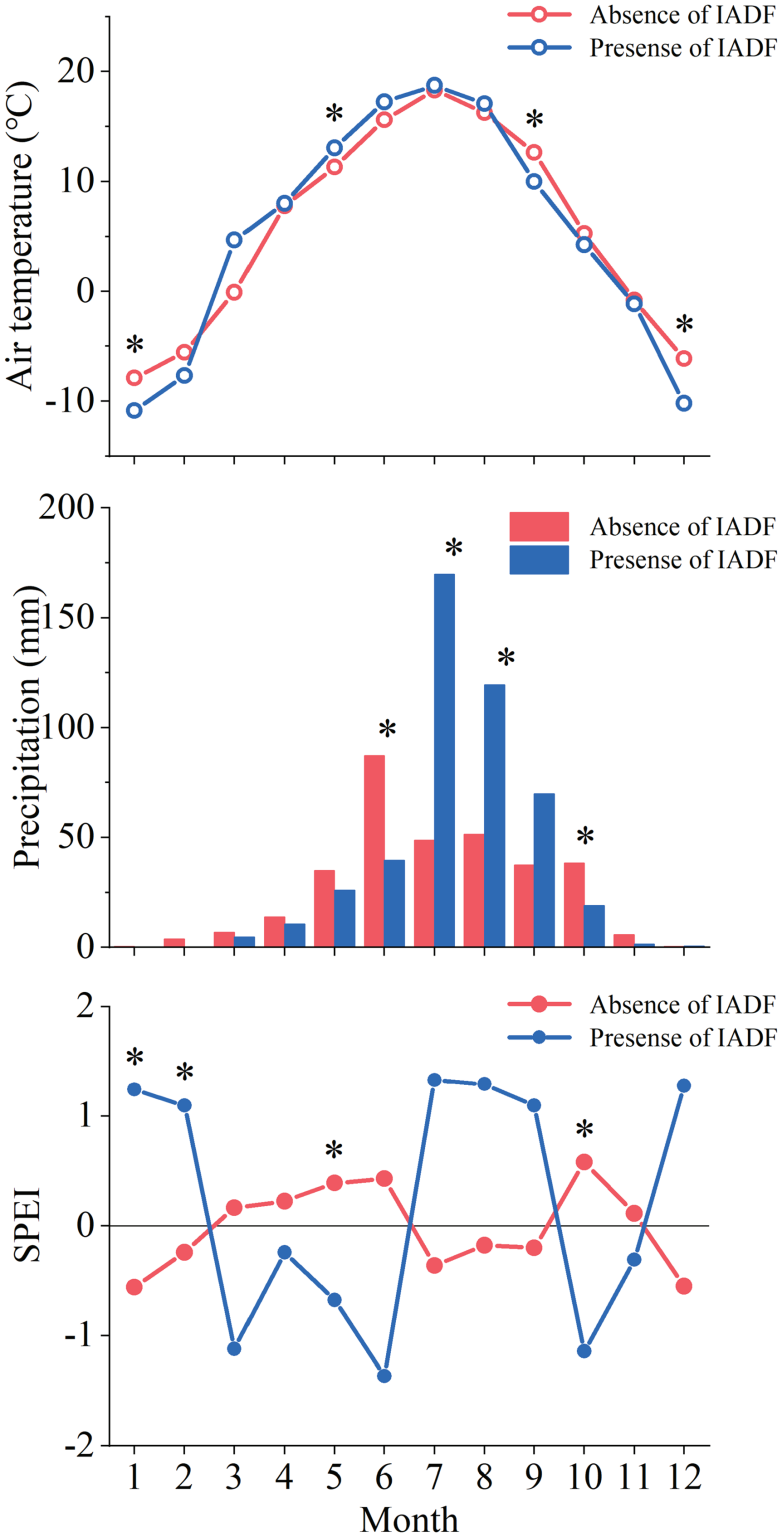
Monthly air temperature, precipitation, and SPEI between absence and presence of IADF during 2017–2019 at three sites. Asterisks represent significant differences (*p*<0.05) between the two categories.

May temperature was significantly higher at the sites where IADF occurred (Figure 5). Lower winter temperature was recorded at sites where IADF occurred. IADF was associated to lower precipitations in June and October, and higher precipitations in July and August. No significant difference in precipitation was observed in the other months. The monthly drought condition between the presence of and absence of IADF was contrary. The climate was dry from March to June and humid from July to September in the IADF group, while it was humid from March to June and July to September in the absence of IADF group.

### Xylem Formation in 2018

**Figure 6:**
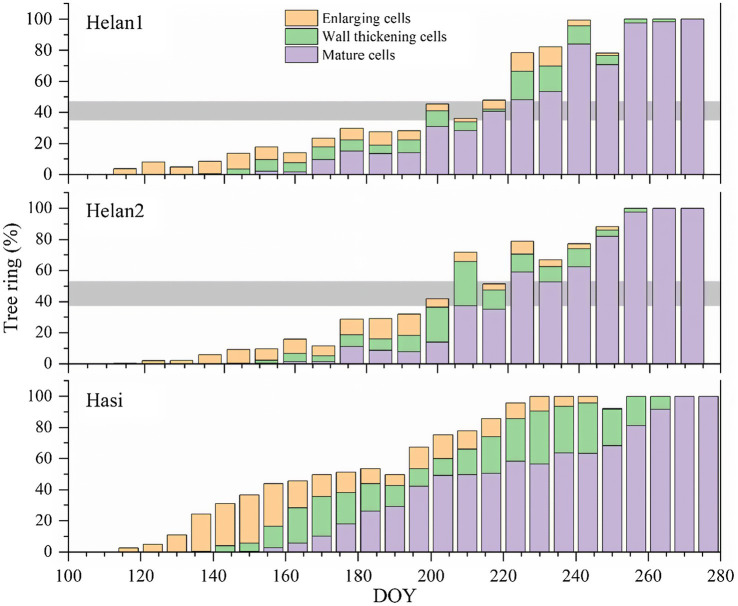
Proportion of tree ring of cells during different phases of xylem formation at three sites in 2018 in a semi-arid region of Northwestern China. Horizontal bars represent IADFs at Helan1 and Helan2.

To test our hypothesis, we focused on the xylogenesis in 2018, the year with IADF formation. The first enlarging cells, corresponding to the onset of xylem cell differentiation, were observed from late April to early May in 2018 (Figure 6). The process of cell enlargement lasted until late August at Hasi, and until mid-September at the two sites in Helan. Compared to Hasi, the number of enlarging cells increased more slowly at Helan1 and Helan2 at the beginning of the growing season. Cell wall thickening began in late May to early June and was completed in mid-September. The first mature xylem cells were observed in mid-June. Xylem differentiation, including cell enlargement and secondary wall formation, lasted for 112, 127 and 130days at Helan1, Helan2 and Hasi, respectively. The total number of xylem cells increased slowly before DOY 200, with only 40% of the xylem cells being produced at the two sites in Helan.

### IADF Phenology

**Figure 7:**
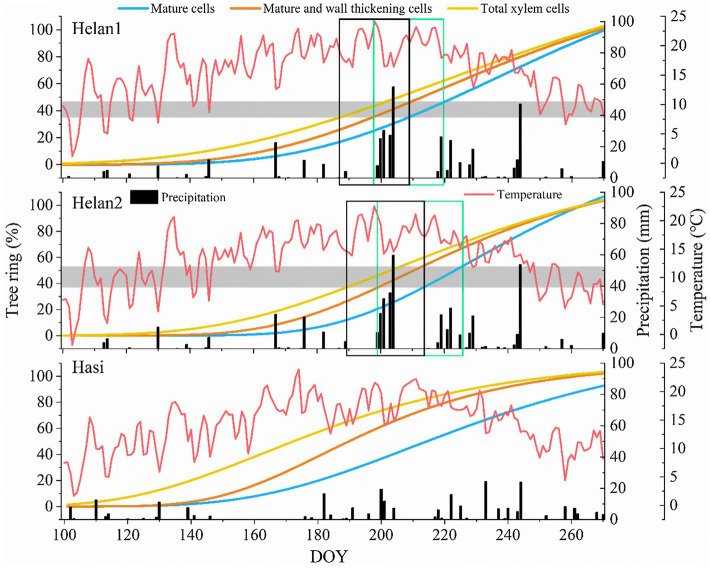
Xylem formation described by Gompertz functions and weather at three sites in 2018 in a semi-arid region of Northwestern China. Horizontal bars represent IADFs at Helan1 and Helan2. Black and green rectangles correspond to the time windows of enlargement and cell-wall thickening of IADF cells.

At the beginning of the growing season, xylem formation progressed slowly under low or absent precipitations at Helan1 and Helan2 (Figure 7), and only 35–37% of xylem cells were formed before DOY 190. IADF cells enlarged between DOY 190 and 210 at the Helan sites. In this period, Helan was warm, and precipitation was absent during the first half of the period when IADF cells enlarged. After DOY 200, the temperature decreased and precipitation recovered. IADF cells underwent wall thickening during DOY 200–220, under abundant rainfall. During IADF formation, the proportion of enlarging cells in the tree ring decreased, and only 12–16% of the tree ring belonging to IADF was formed. After DOY 220, cell number increased again, and more than 50% of the total amount of annual xylem cells was produced within 50days. Regarding at Hasi site, precipitation during DOY 100–150 was three times higher compared to Helan, and 68% of the tree ring was produced before DOY 190. At that date, all the earlywood cells had completed cell enlargement at Hasi, which indicated a faster progression of xylem formation compared to Helan. No IADF cells were observed at Hasi, where cell enlargement of latewood cells progressed after DOY 190.

## Discussion

Earlywood intra-annual density fluctuation is a response to a drought event occurring during the growing season, mainly in the summer (Campelo et al., 2007, 2015). In this study, we investigated the dynamics of xylogenesis and IADF formation in Chinese pine (*Pinus tabuliformis*) in a semi-arid region of northwest China and found that IADF cells were produced under a prolonged summer drought and differentiate during the precipitation deficit in June and abundant rainfall in July and August. These findings suggest that summer drought triggers cell enlargement and affects IADF formation, which allowed us to accept our initial hypothesis.

### IADF Formation and Its Environmental Control

Latewood-like IADF cells enlarged during the drought event, although some of them completed enlargement at the beginning of the abundant rainfall (DOY 190–210). IADF cells started wall thickening when the precipitations had started again (DOY 200) and matured before mid-August. A significant decrease in the number of cambial and enlarging cells at the end of the drought was also observed, suggesting that drought inhibited cambial division and thus limited cell differentiation. Water availability is the primary climatic factor limiting stem growth in dry and warm regions (Rossi et al., 2014). Prolonged and severe summer droughts or high transpiration rates can disrupt the steady-state of water transfer in xylem cells and cause declines in water potential of the xylem (Bogeat-Triboulot et al., 2007). A low turgor pressure during enlargement prevents differentiating tracheids to increase in diameter, resulting in narrow, latewood-like cells (Steppe et al., 2015). Our finding is consistent with other studies, demonstrating that narrower cells are formed under water deficit occurring during formation (Rossi et al., 2009; Battipaglia et al., 2010). According to our findings, some IADF cells completed enlargement during the rainfall period. This unexpected result may represent the time lag between rainfall and the increase of water potential in xylem cells, or be related to a statistical error due to the weekly sampling used in this study.

IADF cells started secondary wall formation during the rainfall period, producing thick cell walls. It is suggested that wall thickness is encoded during the previous phase of cell enlargement (Cuny et al., 2014; Buttὸ et al., 2019). It is widely documented that carbohydrate in the stem, including structural C, such as cellulose and lignin, often accumulates under drought (Muller et al., 2011), suggesting that more carbon is available for the wall thickening process. Furthermore, the amount of materials of wall deposition stays almost constant in most of the cells in a tree ring (Cuny et al., 2014). The reduction in size of IADF cells would lead to more wall materials allocated to wall thickening, producing thicker walls in IADF cells. The availability of soluble sugars regulates the wall deposition process (Cartenì et al., 2018). Our results indicate that climate drivers are uniform for cell enlarging and wall thickening of IADF cells: large IADF cells with thick walls were mainly induced by summer drought. Stangler et al. (2021) demonstrated that the transition from a dry June to a wet July initiated IADF formation in Norway spruce at lower elevations in Germany. Accordingly, we detected the specific IADF phenology under water deficit and showed evidence of the climate driver of enlargement and wall thickening during IADF formation.

### The Functional Explanation of the IADF Formation

IADF formation is a specific adaptation under summer drought. This modification in the xylem structure protects the trees from irreversible damage by xylem cavitation and vessel embolism at the cost of the reduction of water transfer efficiency (Martin-Benito et al., 2013; Puchi et al., 2019). The physiological response to water limitation is to protect trees against irreversible damage when stress becomes severe and long (Claeys and Inzé, 2013). In other words, trees ensure their survival when a water stress is prolonged or becomes more severe.

The rainfall of mid-July improved growing conditions and reactivated cambial activity and cell enlargement. In other words, once water availability is sufficient, xylogenesis recovers the expected activities of cell division and differentiation. During cell enlargement, the vacuoles loaded with sugars increase the turgor pressure and attract water inside the cells, then producing the large earlywood tracheids (Vieira et al., 2020). This mechanism failed under a Mediterranean climate, where a small amount of irrigation in September was unable to trigger a second period of cambial activity (Vieira et al., 2020). The observed flexible xylogenesis reflects the plasticity of our species, which allows trees to survive under water deficit and concentrate growth during favorable water supply conditions. Our study demonstrates the mechanism of IADF formation under summer drought and highlights the plasticity of xylogenesis to the varied water conditions.

### Water Availability and Cell Production

Drought affected both wood anatomy and xylem cell production. Growth dropped once water deficit began. Enlargement of the first earlywood cells occurred during the high precipitation of April-May at Hasi, resulting in 30% of the tree ring. In the same period, only 10% of xylem cells started enlargement at Helan, because of a lack of rain and despite the water provided by the snowmelt in spring. The rainfall in the early growing season at Hasi provided sufficient water availability, thus trees could maintain high photosynthetic rates for sustaining cambium division and xylem growth, processes that are large carbon sinks (Bréda et al., 2006; Zhang et al., 2020). Moreover, the precipitation in April and May might have helped trees to maintain a higher water potential and protect the xylem cells from the negative influence of drought. As a result, xylem cells at Hasi were able to differentiate and mature their secondary walls under dry conditions, thus producing tree rings lacking IADF. Conversely, dry conditions during March–June may limit stem growth and, in case of prolonged lack of rain, accelerate leaf senescence to maintain the water balance. This defense mechanism led to growth losses, as observed by the lower amount of xylem cells produced at Helan, compared to Hasi, before the July precipitations. Our weekly monitoring of xylem formation provides evidence of the limiting effect of drought on cambial activity and xylogenesis at Helan. Dendrochronological studies also demonstrated that water availability of March–July affected stem growth (Li et al., 2007; Cai, 2009). Moreover, the precipitation from late spring to early summer (May–June) played a role in stem growth of *Sabina przewalskii* in semi-arid northwestern China (Gou et al., 2015; Yang et al., 2019, 2021).

## Conclusion

As a structural anomaly of the tree ring, IADF is a suitable indicator of weather fluctuations occurring during the growing season. However, the physiological processes behind IADF formation and the impacts of the climate drivers remain partially unknown. To our knowledge, this is the first time that timings and dynamics of IADF formation have been assessed at high temporal resolutions. We investigated the weekly xylogenesis of Chinese pine at three sites in semi-arid continental China by describing the development of an *E*-IADF. Prolonged and severe drought in June and early July affected the processes of xylem production and differentiation and induced IADF. Specifically, drought limited enlargement and affected wall thickening, resulting in IADF cells with narrow tracheids and wide walls. Rainfall on April–May provided sufficient water availability for xylogenesis, protecting trees against summer drought. Warming-induced droughts may become more frequent and severe during the summer in the drier regions of the northern hemisphere, enhancing the occurrence of IADF in trees. The formation of IADF appears to be a defense mechanism of trees against unfavorable environmental conditions, which ensures plasticity to the conducting system of trees and the capacity to tolerate and resist to water deficits.

## Data Availability Statement

The original contributions presented in the study are included in the article/supplementary material, and further inquiries can be directed to the corresponding author.

## Author Contributions

JG, SR, and BY designed the study and revised the manuscript. JG and SR performed the statistical analysis. JG wrote the first version of the manuscript. All authors contributed to the article and approved the submitted version.

## Funding

This work was jointly supported by the National Natural Science Foundation of China (grant no. 42130511 and 41520104005), and a State Scholarship Fund provided by the China Scholarship Council (202004910757).

## Conflict of Interest

The authors declare that the research was conducted in the absence of any commercial or financial relationships that could be construed as a potential conflict of interest.

## Publisher’s Note

All claims expressed in this article are solely those of the authors and do not necessarily represent those of their affiliated organizations, or those of the publisher, the editors and the reviewers. Any product that may be evaluated in this article, or claim that may be made by its manufacturer, is not guaranteed or endorsed by the publisher.
